# Influence of Posture and Frequency Modes in Total Body Water Estimation Using Bioelectrical Impedance Spectroscopy in Boys and Adult Males

**DOI:** 10.3390/nu6051886

**Published:** 2014-05-05

**Authors:** Masaharu Kagawa, Connie Wishart, Andrew P. Hills

**Affiliations:** 1 Institute of Nutrition Sciences, Kagawa Nutrition University, Saitama 350-0288, Japan; 2 National Institute of Public Health, Saitama 351-0197, Japan; 3 School of Public Health, Curtin University, Western Australia 6102, Australia; 4 Institute of Health and Biomedical Innovation, Queensland University of Technology, Queensland 4059, Australia; E-Mail: c.wishart@qut.edu.au; 5 Mater Research Institute, the University of Queensland and Griffith Health Institute, Griffith University, Queensland 4101, Australia; E-Mail: ahills@mmri.mater.org.au

**Keywords:** body fluid, deuterium, dilution technique, impedance technique, prediction equation, accuracy, technical error

## Abstract

The aim of the study was to examine differences in total body water (TBW) measured using single-frequency (SF) and multi-frequency (MF) modes of bioelectrical impedance spectroscopy (BIS) in children and adults measured in different postures using the deuterium (^2^H) dilution technique as the reference. Twenty-three boys and 26 adult males underwent assessment of TBW using the dilution technique and BIS measured in supine and standing positions using two frequencies of the SF mode (50 kHz and 100 kHz) and the MF mode. While TBW estimated from the MF mode was comparable, extra-cellular fluid (ECF) and intra-cellular fluid (ICF) values differed significantly (*p* < 0.01) between the different postures in both groups. In addition, while estimated TBW in adult males using the MF mode was significantly (*p* < 0.01) greater than the result from the dilution technique, TBW estimated using the SF mode and prediction equation was significantly (*p* < 0.01) lower in boys. Measurement posture may not affect estimation of TBW in boys and adult males, however, body fluid shifts may still occur. In addition, technical factors, including selection of prediction equation, may be important when TBW is estimated from measured impedance.

## 1. Introduction

Body composition, including fat mass (FM) and fat-free mass (FFM), is an important variable in the assessment of health status. Obesity has been defined as a state of excessive fat deposition [1,2] and the assessment of body composition assists in identifying individuals with metabolic risks. In addition, while body mass index (BMI: kg/m^2^) and other simple anthropometric indices have been utilized as convenient screening tools for obesity, assessment of body composition reduces misclassification of individuals at risk.

Body composition can be determined using a wide range of techniques. Each technique varies not only in its accuracy and precision, but also in cost, portability, convenience, and requirements for accredited operators. Bioelectrical impedance analysis (BIA) is one of the most commonly utilized techniques as it is simple, portable and cost- and time-efficient. The technique assesses differences in the electrical conductivity between tissues. Tissues that contain water and electrolytes have higher conductivity compared to those with less body fluid. From the measurement of electrical conductivity, resistance (R) and reactance (Xc) can be determined. These components can be utilized to calculate impedance (Z) based on their association Z^2^ = R^2^ + Xc^2^ and also a phase angle based on a ratio of Xc to R [3]. In addition, together with information on the length (L) or height (Ht), a total volume of body water (TBW) can be determined [4,5]. Furthermore, while R has been used most frequently, R, Xc, and Z have been used to estimate TBW, intra-cellular fluid (ICF) and extra-cellular fluid (ECF) as well as percentage body fat (%BF) of individuals [4].

Existing BIA devices can be divided into single-frequency BIA (SFBIA), multi-frequency BIA (MFBIA) and bioelectrical impedance spectroscopy (BIS). SFBIA devices generally use a frequency of 50 kHz that passes through both ECF and ICF [4]. In comparison, MFBIA uses multiple frequencies in the range of 1 to 1000 kHz and enables one to distinguish between ICF and ECF. A previous study reported that a low frequency, generally below 20 kHz, is used to predict ECF whereas a higher frequency (above 50 kHz) is used to estimate TBW in MFBIA [6]. As a result, ICF can be determined from the difference of the two. Although it has been suggested that MFBIA may overestimate %BF of lean individuals and underestimate that of obese individuals [7], error in estimation of %BF may be minimized compared with SFBIA [8]. BIS is a more sophisticated model that uses a wide range of frequencies and non-linear mathematical algorithm to assess relationships between R and body fluid. This allows estimation of R extrapolated to zero (R_0_) and infinite (R_∞_) frequencies and development of empirically-derived prediction equations [4,5,9]. Although both accuracy and precision of results may vary depending on the characteristics of the study population [9], past studies have reported that BIS provides better estimation of ECF than SFBIA [4,5,10,11] and also has an acceptable accuracy and precision using an animal model [12]. In addition, a technique known as “segmental BIA” is available which determines information on total body composition through measurements of each segment (*i.e.*, upper and lower limbs and the trunk). A previous review has described a number of advantages and considerations [13], and another study reported that segmental BIA can provide valid information on body composition compared with the four-compartment model [14]. However, most studies have been undertaken on adults and studies of children are relatively scarce. Consequently, little knowledge is available on any differences in TBW estimation between adults and children and the effects of using different frequencies.

In addition, wide variations in measurement posture are commonplace using the impedance technique depending on the device used. Many hand-to-foot models measure in a supine position, the posture recommended in the European Society for Parenteral and Enteral Nutrition (ESPEN) guidelines [10]. However, modern segmental BIAs that are in a scale type are designed to measure in a standing position [13]. A previous study on the influence of posture during measurements reported 2%–5% changes in ECF and 1.8%–8.0% changes in ICF depending on body position [15]. Another study reported no significant differences in %BF using a hand-to-foot device but a significant increase in %BF using a hand-to-hand device [8]. These results suggest that body posture may influence estimation of body fluid status and therefore estimation of %BF using the impedance technique. However, these studies were conducted on adults and no previous research has reported differences between adults and children.

Therefore, the present study aimed to examine influences of frequency modes and measurement posture in estimation of TBW in adult males and boys. The estimated TBW was compared with the result obtained from the reference deuterium (^2^H) dilution technique.

## 2. Experimental Section

The study was approved by the Human Research Ethics Committee of Queensland University of Technology and adhered to the principles of medical research established by the National Health and Medical Research Council [16]. Boys aged below 15 years or adult males aged above 20 years with no medical conditions or under medication were included in the study. Participants were recruited through flyers. All participants were given an information package and all signed a consent form prior to their participation. For participants below 18 years of age, parents or legal guardians also signed the consent form. All participants were provided with a $20 gift voucher after their full participation in the study. In total, 49 participants including 23 boys aged between 6 and 14 years and 26 adult males aged between 23 and 82 years, completed all assessments and were included in the study. All participants were instructed to fast overnight and void their bladders in the morning, prior to measurements being conducted. All assessments on children were conducted by the primary investigator.

### 2.1. Anthropometry

All participants underwent measurements of stature using a stadiometer to the nearest 0.1 cm and body weight using a digital weighing scale to the nearest 0.1 kg. Waist circumference was measured at the narrowest point between the 10th rib and the iliac crest using a steel anthropometric tape. All measurements were conducted according to the protocol of the International Society for the Advancement of Kinanthropometry (ISAK) [17]. All measurements were taken by a level three anthropometrist accredited by ISAK with an intra-tester technical error of measurements (TEM) of less than 1.0%, below recommended levels [18,19]. From these measurements, BMI and waist-to-height ratio (WHtR) were calculated.

### 2.2. Bioelectrical Impedance Spectroscopy (BIS)

Body fluid status was determined using a BIS device (Imp SFB7, ImpediMed Ltd, Brisbane, Australia) with functionality to switch between multi-frequency (MF) and single-frequency (SF) modes. In the MF mode, TBW was estimated using a wide range of frequencies between 4 and 1000 kHz with 256 data points [20]. In the SF mode, two of the five fixed frequencies (*i.e.*, 50 kHz and 100 kHz) of the device that are commonly selected were used [20].

The device was calibrated before measurements of each participant. All participants rested (lying on a bed) for at least five minutes prior to the measurement. Electrodes were placed on the dorsal surface of the wrist and the ankle as well as at the base of the second or third metacarpal-phalangeal joints of hand and foot after the skin was cleaned with an alcohol wipe. The lead wires were attached to the appropriate electrodes and participants were instructed to abduct their limbs from the trunk. After measurement in the supine position, participants were instructed to stand up and stay in the same position for at least five minutes before measurement in the standing position. The measurements were repeated in both positions after staying in the position at least five minutes. Length of rest time was consistent with the protocol used in a previous study [21]. While the participant was in the same posture, triplicate measurements were conducted and a median value determined. After completion of two sessions of triplicate measurements at each measurement posture, an average of two median values was calculated for each variable.

From each measurement, TBW, ECF, ICF, FFM and FM were calculated for the MF mode using built-in algorithms. For the SF mode, Xc and R were recorded for each frequency. Similar to a previous study [22], all measurements were conducted with resistivity coefficients of 273.9 for zero/extracellular (ρ_e_) and 937.2 for infinite/intracellular (ρ_i_), body density of 1.05 g/cm^3^ and body proportion of 4.3. From the obtained FM and FFM results, %BF was calculated for the MF mode. For the SF mode, Z was calculated from mean Xc and R using an equation √(R^2^ + Xc^2^). Calculated Z values were then utilized to estimate TBW of the study groups using age-, gender-, and frequency-specific prediction equations. In the present study, estimation of TBW for boys was conducted using an equation by Davies *et al.* [23] with Z determined from a frequency of 50 kHz (Z_50_):

TBW = 0.6 × (Ht_2_/Z_50_) − 0.5 (1)

For males, equations by Deurenberg *et al.* [24] were used to estimate TBW using Z determined from frequencies of 50 kHz (Z_50_) and 100 kHz (Z_100_):

TBW = 6.53 + 0.3674 × Ht_2_/Z_50_ + 0.17531 × body weight − 0.11 × age + 2.83 (2)

TBW = 6.69 + 0.34573 × Ht_2_/Z_100_ + 0.17065 × body weight – 0.11 × age + 2.66 (3)

### 2.3. Deuterium Dilution Technique

Deuterium (^2^H) dilution technique was the reference method used for TBW. Prior to the assessment, a 10% deuterium oxide (D_2_O) solution was prepared by mixing 100 mL of 99.9% D_2_O solution (Aldrich Chemistry, Sigma-Aldrich Pty Ltd, Sydney, Australia) and 900 mL of tap water. The solution was sterilized at 120 °C for 10 min using an autoclave. After collecting a pre-dose urine sample, the body weight of participants was measured using a weighing scale. The dose amount of 10% D_2_O solution was calculated as 0.5 × body weight (kg) and weighed using a scale. All participants consumed the weighed 10% D_2_O solution and re-weighed the drinking cup to record the precise amount consumed. After consumption of the 10% D_2_O solution, participants were instructed to collect a post-dose urine sample after five hours, to ensure that equilibration of ^2^H within the body fluid pool was reached.

Both pre- and post-dose samples were analyzed using an isotope ratio mass spectrometry (IRMS: Hydra 20-20, SerCon Mass Spectrometry, SerCon Limited, Cheshire, CW1 6YY UK). All analyses were conducted at the laboratory at the Institute of Health and Biomedical Innovation (IHBI) of Queensland University of Technology (QUT) in Brisbane, Australia. TBW was calculated using the following equation:

TBW (kg) = ((*W* × *A*/*a*) × (ΔDD/ΔBW))/(1000 × 1.041) (4)

where *W* = total weight of water added when making the dose dilution (g), *A* = weight of dose taken by the participant (g), *a* = weight of dose in diluted dose (g), ΔDD = enrichment of ^2^H in the diluted dose (ppm excess ^2^H), and ΔBW = enrichment of ^2^H in body water (ppm excess ^2^H). The value of 1.041 was based on an assumption that the dilution space or the volume of the distribution of ^2^H is 1.041 times greater than TBW [25].

All statistical analyses were conducted using the PASW^®^ Statistics package (version 18.0.0, IBM, Chicago, IL, USA). Paired *t*-tests were conducted to compare results obtained from different postures (*i.e.*, supine and standing positions). In addition, TBW estimated from different frequency modes were compared with the results from the dilution technique using repeated measures of analysis of variance (ANOVA) and a Bonferroni *post hoc* test. Results were expressed as mean ± standard error (SE). In addition, variability of estimated TBW for the study population from different frequency modes were determined using correlation coefficients, limits of agreement (*i.e.*, difference ± 1.96 × standard deviation), and the Bland and Altman plots [26] using the dilution technique as the reference. All statistical tests used significant level of 0.05 unless otherwise stated.

## 3. Results

Physical characteristics of the participants were 9.8 ± 0.5 years, 144.1 ± 3.2 cm and 36.6 ± 2.4 kg for boys and 36.9 ± 2.7 years, 174.3 ± 1.4 cm, and 76.5 ± 3.2 kg for adult males, respectively (Table 1). WHtR, an index of abdominal fat accumulation with the cut-off point of 0.5, were 0.44 ± 0.01 in boys and 0.49 ± 0.01 in adult males, respectively.

**Table 1:** Demographic characteristics of participants.

	Boys ( *n* = 23) Mean ± SE	Males ( *n* = 26) Mean ± SE
**Age (years)**	9.8 ± 0.5	36.9 ± 2.7
**Stature (cm)**	144.1 ± 3.2	174.3 ± 1.4
**Body weight (kg)**	36.6 ± 2.4	76.5 ± 3.2
**BMI (kg/m^2^)**	17.2 ± 0.5	25.0 ± 0.8
**WHtR**	0.44 ± 0.01	0.49 ± 0.01

Table 2 shows differences in results obtained from supine and standing positions. In both adult males and boys, there were no significant differences in TBW using the MF mode in supine and standing positions. There were no differences in estimated FFM, FM and %BF between measurement postures using the MF mode. Adult males, however, showed a statistically significant (*p* < 0.05) difference in TBW in independent measurement sessions in the supine position (44.3 ± 1.5 L and 44.2 ± 1.5 L). ECF measured in the standing position was significantly (*p* < 0.01) increased compared with the supine position in both groups (males: 19.2 ± 0.6 L *vs.* 18.9 ± 0.6 L, boys: 10.0 ± 0.6 L *vs.* 9.7 ± 0.6 L). There was also a significant (*p* < 0.01) decrease in ICF in the standing position in both groups. Using the SF mode, while no difference in Z_50_ was observed between measurement postures in males, a significant decrease in Z_50_ was observed in the standing position in boys (665.3 ± 16.1 Ω in the supine position *vs.* 656.9 ± 16.3 Ω in the standing position, *p* < 0.01). A similar result was observed using 100 kHz (*p* < 0.05 in males and *p* < 0.01 in boys). In addition, adult males showed a significant difference in Z values in each supine measurement, boys showed significantly different Z values from standing sessions (data not shown).

**Table 2:** Differences in variables between measurement postures in boys and adult males.

		Boys (*n* = 23) Mean ± SE			Adult Males (*n* = 26) Mean ± SE		
Posture		Supine	Standing	*p*-value	Supine	Standing	*p*-value
**Multi-Frequency**	TBW (L)	21.3 ± 1.4	21.2 ± 1.5	0.348	44.3 ± 1.5	44.2 ± 1.5	0.823
	ECF (L)	9.7 ± 0.6	10.0 ± 0.6	<0.001	18.9 ± 0.6	19.2 ± 0.6	< 0.001
	ICF (L)	11.6 ± 0.9	11.3 ± 0.8	0.005	25.4 ± 0.9	25.0 ± 0.9	0.005
	FFM (kg)	29.1 ± 2.0	29.0 ± 2.0	0.353	60.5 ± 2.0	60.4 ± 2.1	0.819
	FM (kg)	7.4 ± 0.8	7.6 ± 0.8	0.353	16.0 ± 1.4	16.0 ± 1.5	0.824
	%BF (%)	20.1 ± 1.3	20.5 ± 1.3	0.340	20.1 ± 1.2	20.1 ± 1.3	0.820
**Single-Frequency**	Z_50_ (Ω)	665.3 ± 16.1	656.9 ± 16.3	<0.001	473.1 ± 8.9	472.2 ± 9.4	0.524
	Z_100_ (Ω)	635.0 ± 15.8	626.5 ± 16.2	<0.001	447.5 ± 8.6	444.3 ± 9.0	0.021

Using repeated measures ANOVA, the present study also examined the accuracy of the estimated TBW from the impedance technique using the dilution technique as the reference (Table 3). In boys, TBW values estimated from the MF mode were comparable to the results from the dilution technique regardless of the measurement postures. However, in males, the MF mode provided a significantly (*p* < 0.01) greater TBW in both postures compared to results from the dilution technique. When TBW was estimated using the SF mode with a selected prediction equation, no significant difference with the result from dilution technique was observed in males regardless of their measurement posture and frequency used. However, TBW estimated from boys using a frequency of 50 kHz was significantly (*p* < 0.01) smaller compared to the result from the dilution technique, regardless of their measurement posture.

**Figure 1:**
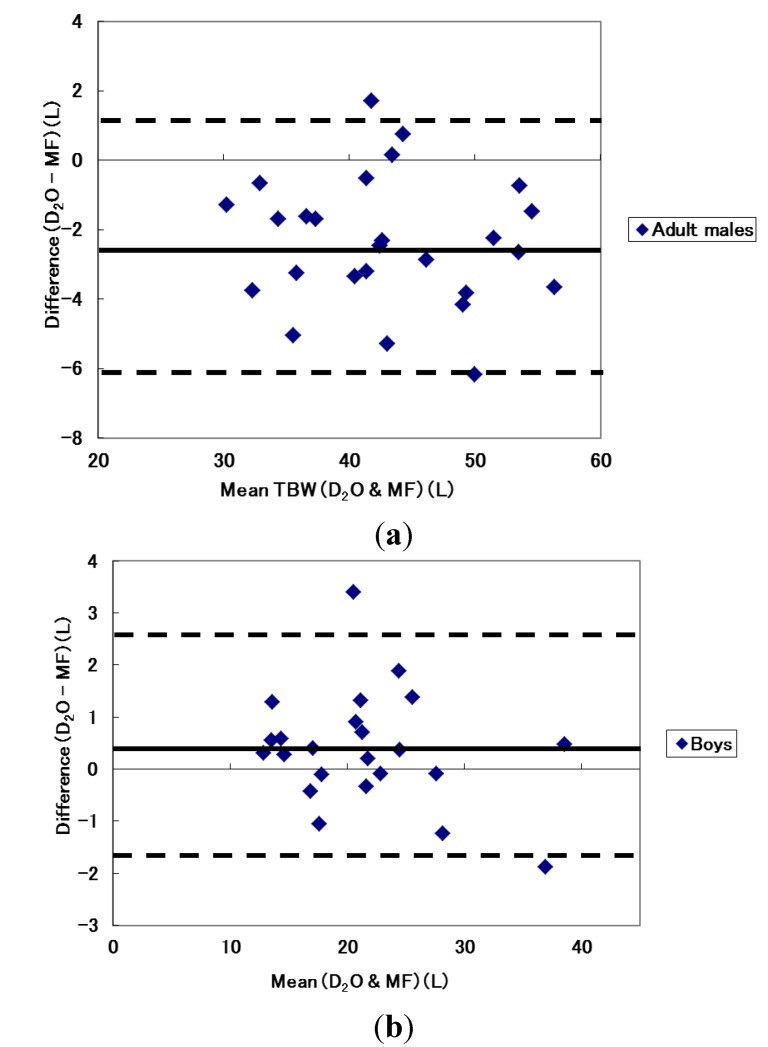
Bland and Altman plots between TBW estimated from the dilution technique and multi-frequency mode for (**a**) adult males and (**b**) boys.

**Figure 2:**
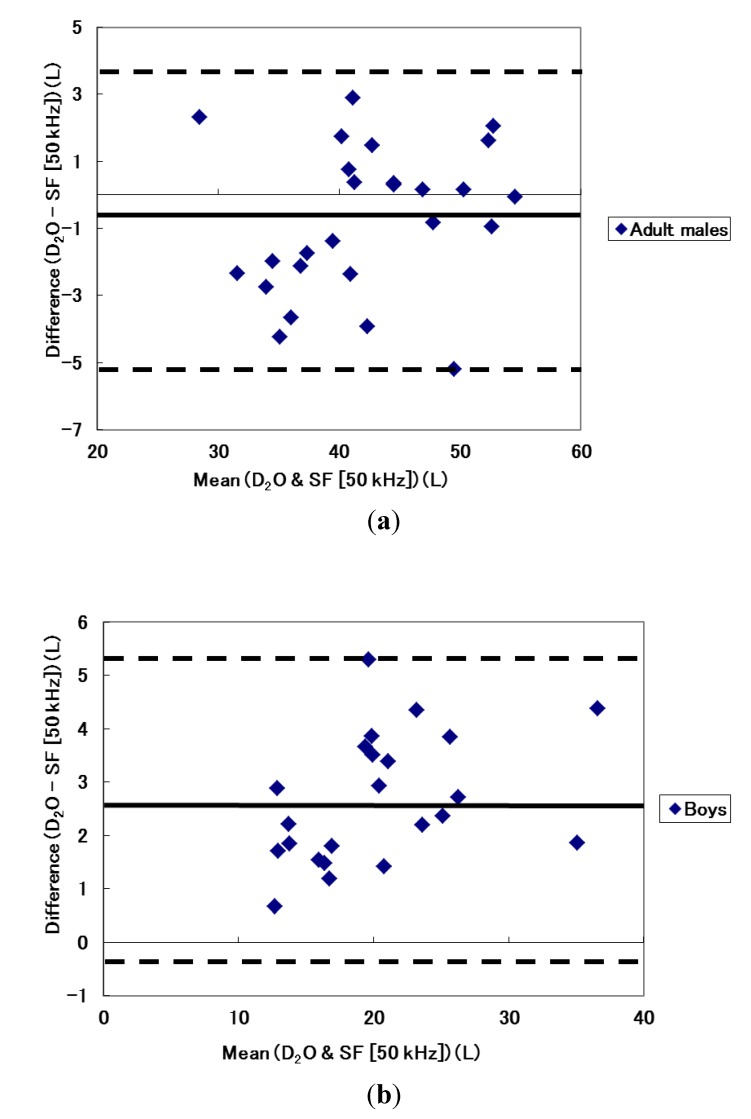
Bland and Altman plots between TBW estimated from the dilution technique and single-frequency mode (50 kHz) for (**a**) adult males and (**b**) boys.

The variability of TBW estimation using different frequency modes are shown in Table 4. Compared with the dilution technique, results from both MF and SF modes showed high correlation coefficients of 0.956 to 0.988. However, Bland and Altman plots for the supine position showed that TBW of almost all adult males was overestimated when the MF mode was used (Figure 1a) and calculated limits of agreement indicated an average of 2.4 L overestimation with a wide variability of about 3.7 L (Table 4). In comparison, boys showed relatively accurate estimation of TBW but some boys with a larger TBW were overestimated (Figure 1b). Calculated limits of agreement indicated that TBW estimation using the MF mode for boys had an underestimation of 0.38 L with variability of about 2 L. Similarly, TBW from the dilution technique and the SF mode using 50 kHz was compared. In males, the Bland and Altman plot indicated that individuals with relatively low TBW values (less than 40 L) were overestimated by the SF mode whereas the opposite was true for those with relatively high TBW (greater than 40 L). Limits of agreement indicated, on average, overestimated about 0.7 L with a variability of approximately 4 L. In boys, TBW was underestimated in all participants with potentially greater underestimation in individuals with a higher TBW (Figure 2). Limits of agreement showed about 2.7 L of underestimation with a variability of 2.4 L.

**Table 3:** Differences in TBW estimated from different techniques.

	Boys ( *n* = 23) Mean ± SE		Males ( *n* = 26) Mean ± SE	
TBW_(2H dilution)_ (L)	21.7 ± 1.4		41.9 ± 1.5	
**Supine**				
Device	Mean ± SE	*p*-value	Mean ± SE	*p*-value
Multi-Frequency (L)	21.3 ± 1.4	0.333	44.3 ± 1.5	<0.001
Single-Frequency Z_50_ (L)^ †^	19.0 ± 1.3	<0.001	42.6 ± 1.4	0.566
Single-Frequency Z _100_ (L) ^‡^	-	NA	42.2 ± 1.3	1.000
**Standing**				
**Device**	**Mean ± SE**	***p*-value**	**Mean ± SE**	***p*-value**
Multi-Frequency (L)	21.2 ± 1.5	0.250	44.2 ± 1.5	<0.001
Single-Frequency Z_50_ (L) ^†^	19.3 ± 1.3	<0.001	42.7 ± 1.4	0.461
Single-Frequency Z _100_ (L) ^‡^	-	NA	42.4 ± 1.4	1.000

^†^ TBW was estimated using the estimation equation Deurenberg *et al.* [24] for males and Davies *et al.* [23] for boys; ^‡^ TBW was estimated using the estimation equation by Deurenberg *et al.* [24].

**Table 4:** Variability of TBW estimation using different frequencies compared with the dilution technique.

		Boys (*n* = 23)	Males (*n* = 26)
Multi-Frequency	Correlation coefficient	0.988	0.970
	Limits of Agreement	0.378 ± 2.14 (2.518, −1.762)	−2.361 ± 3.655 (1.29, −6.015)
Single-Frequency Z_50_^†^	Correlation coefficient	0.985	0.956
	Limits of Agreement	2.6524 ± 2.357 (5.009, 0.295)	−0.7465 ± 4.289 (3.543, −5.036)

^†^ TBW was estimated using the estimation equation Deurenberg *et al.* [24] for males and Davies *et al.* [23] for boys.

## 4. Discussion

The present study investigated the influence of posture and frequency modes of impedance technique in the estimation of TBW in adult males and boys. Results confirmed that measurement posture had no significant influence on TBW estimation and therefore no influence on estimation of body composition in this convenience sample. The results were consistent with earlier reports using SF devices [21]. This suggests that influence of body posture during measurements using impedance technique has a minimal impact on overall estimation of body composition. However, the present study showed significant changes in ICF and ECF volumes depending on posture during measurements in both adult males and boys. It has been suggested that a change in posture will cause redistribution of ECF. The observed results, although smaller in magnitude, were consistent with a previous study that reported change of ECF and ICF volumes by posture [15]. The result was inconsistent with another study that reported a redistribution of ECF only occurred between body segments (*i.e.*, the limbs and the trunk) with total ECF volume not altering as a function of change in measurement posture [27]. The present findings of change in ECF and ICF with no overall change in TBW may be due to a larger sample size compared to the previous study that examined only 11 males.

In addition to a possible fluid shift as a result of change in measurement posture, the presence of stray capacitance may also have influenced the observed outcomes. Weyer and colleagues [28] suggested that of the two fundamental stray capacitances in the impedance technique, the one formed between the human body and the ground may have considerable impact on the reading. Technical factors such as stray capacitances as well as variables such as body proportions, body density, and resistivity coefficients may be important in interpreting accuracy and quality of results. Boys showed a significant decrease in Z_50_ measured in the standing position. Since the frequency of 50 kHz can go through both ECF and ICF, a reduction in Z may indicate a reduction in TBW. However, results from the MF mode did not show a change in TBW between postures. As no differences in Z_50_ were observed from males, this may suggest that, together with body fluid shift some technical factors influenced measurements in boys.

While no specific pattern was observed from the MF mode, estimation of TBW in adult males using the SF mode showed a different pattern depending on whether the participant had TBW greater or lesser than 40 L. Compared to adult males, boys showed a better correlation and agreement between the dilution technique and the impedance technique (both SF and MF modes). A smaller difference in results from the dilution technique and both MF and SF modes and narrower limits of agreement indicates the accuracy of the impedance technique. Observed differences in TBW estimation may be associated with a number of technical factors, such as resistivity coefficients, a body density, a body proportion factor and also prediction equations to estimate TBW using measured Z for the SF mode. The current study used default values for resistivity coefficients, a body density and a body proportion that were derived from healthy Caucasian adults. Although other studies have adopted the same default settings in unhealthy populations (e.g., obese) [22] or children [29,30], the estimation of TBW or Z values in the current study, particularly in boys, may be the result of technical error. In addition, the estimated TBW from a frequency of 50 kHz showed greater underestimation or noticeable pattern in both males and boys. This may be explained by application of prediction equations to estimate TBW. In this study, TBW for males was estimated by using the equation by Deurenberg *et al.* [24] and TBW of boys were calculated using the equation by Davies *et al.* [23] that was derived from a small group of children (*n* = 26). While the equation by Deurenberg *et al.* [24] was derived from 139 healthy volunteers, the equation by Davies *et al.* [23] was derived from a group of children with particular health conditions, including growth hormone deficiency, inflammatory bowel disease and diabetes. In addition, while the equation was derived from both boys and girls, the equation does not include gender as a variable. Although the age range was matched with the sample of the present study, it may be possible that application of these equations may also affect accuracy and variability of the results. These possibilities suggest the importance of considering the abovementioned technical issues in differentiating biological influence such as fluid shift caused by a change in a measurement posture and also to improve the accuracy of the results, particularly using the SF mode.

## 5. Conclusions

In summary, the present study clarified that estimation of TBW using the MF mode of BIS device is not affected by measurement posture regardless of participants’ maturational status or body size. Accordingly, estimation of body composition, including %BF is not affected by change in measurement posture. However, it should be noted that change in posture may be associated with fluid shift within the body that may alter values for ECF, ICF and Z. In addition, it is important to consider technical factors associated with measurements, including stray capacitances, resistivity coefficients, body proportion factor and also selection of appropriate prediction equations in order to differentiate the effect of measurement posture and technical error. As information on appropriate resistivity coefficients and body proportion factors for children is very scarce future research should consider explore appropriate values for this population. Similarly, as the current study was based on a relatively small sample size, future research should replicate the study using a larger group in different age categories as well as including females to examine gender differences.
